# Measurement of Patient's Perception Levels With Reference to Physician’s Empathy: Private Hospitals Scenario

**DOI:** 10.7759/cureus.18684

**Published:** 2021-10-11

**Authors:** Alpaslan Mert, Ayşegül Kaptanoğlu, Emir Hasan Olmez

**Affiliations:** 1 School of Health Sciences, Beykent University, Istanbul, TUR; 2 Department of Health Management, School of Health Sciences, Beykent University, Istanbul, TUR; 3 Beykent Vocational High School, Beykent University, Istanbul, TUR

**Keywords:** doctors’ empathy, patients’ perception, physician, health, perception levels

## Abstract

Purpose

This study aims at understanding the empathy of doctors and the perception level of patients about it. The survey was conducted including doctors and patients who were cured by these doctors.

Methods

Patient’s Perception Scale of Physician Empathy and Jefferson Scale of Physician Empathy were applied to 70 physicians who worked at 35 private hospitals in Istanbul and 420 patients who received health services from these physicians. Statistical Package for the Social Sciences (SPSS) 24.0 program (IBM Corp., Armonk, NY) was used for statistical analysis.

Results

Physicians who worked in the internal medicine department had more empathy toward the patients. Physicians who were new to the medical profession or who were young had higher empathy levels. Patients who received health services perceived that the surgeons had more empathy. Moreover, it was found that the empathy perception among the educated patients was significantly higher towards physicians.

Conclusion

The perception level of patients for empathy depends directly on the empathy developed by the doctors. Providing better working conditions for the physicians and preparing the educational plans to increase the health information of patients could improve the physician's empathy with the patients and patient will get better health services.

## Introduction

Empathy is the most important humanistic part of patient care and symbolizes healthcare and professionalism in medicine at its best [1]. There is constant communication between physicians, other healthcare professionals working in the health sector, and patients receiving treatment services or their relatives. While communicating with patients, physicians should have a humanitarian attitude towards them and must achieve empathy. Empathic engagement of physicians with patients has been shown to be beneficial for the patients in terms of compliance, positive clinical outcomes, and fewer complications [2,3]. Empathy holds a lot of importance in the context of patient care and could be described primarily as a cognitive attribute that involves the experiences of the patients, their concerns, and perspectives along with the ability to communicate this understanding and an intention to help. This is not a one-way benefit; it also helps physicians against their professional stress and burnout, thereby benefiting their well-being [4].

In the context of scientific data and pathological findings, treatment regimens are decided for the patient. This raises the question that when medicines cure a disease, what is the role of empathy shown by the physicians, and how the patient's perception of the physician is important for a treatment process? Physicians who adapt to the current status of feelings of their patients could reciprocate with them in an appropriate manner and do what is suitable for the patients understanding their state of mind. Based on these observations, physicians could define ways to calm fears, anger or brighten the mood of the patients [5].

For this reason, it is thought that basic information about communication and empathy should be given to our physicians during their medical education by psychiatrists. Indifferent to the gender, empathy helps the doctor to understand his patient better and this helps him in finding the right course of action specific to a person or situation. Positive interaction between patient and doctor increases the feeling of trust, appreciation, and respect. Based on the development of these feelings, physicians could inspire their patients, affect their behavior, and increase their motivation in a much better way [6].

In spite of the significance of empathic engagement of physicians with their patients in patient care, the research conducted to link it with patient outcomes and other aspects is scarce. There are many aspects that need to be deciphered about empathy with reference to the patients as well as physicians such as if there exists any significant relationship between physician empathy and patients' perceived empathy scores. There might be a difference in empathy scores based on the specialization of the physician as well as gender which we have explored in the current study. We further tried to establish, if any relationship exists between the empathy scores of physicians and their ages along with the duration of the professional experience. This study has also focussed on the effect of the specialization branch under which patient is being treated on the patient perception of the physician's empathy and if patients' perceived empathy scores differ significantly according to their ages and gender.

Education and professional life have a direct effect on the thinking ability of an individual, and therefore, the effect of both of these factors was also studied on patients' perceived empathy scores and profession.

## Materials and methods

Research model

We have used the relational screening models, one of the general scanning model to examine the relationship between the perception of the patient and the physician's empathy. Studies using scanning models are those which aim to depict a past or present event or situation without intervening or changing it as it is. Relational screening model is a research method that examines the existence of a relationship between one or more variables and the level of this relationship [5].

Measuring tools

"Physician Empathy Scale" and "Patient Perception Scale on Physician Empathy" were used as measurement tools. Both are 7-point Likert scale with strongly disagree as "1," partially disagree "2," disagree "3," undecided "4," partially agree "5," agree "6" and strongly agree "7." Physician Empathy Scale consists of 20 items in total and Patient Perception Scale on Physician Empathy consists of 10 items. Both scales were analyzed in one dimension over the total score.

The validity and reliability study of the Patient Perception Scale on Physician Empathy was conducted within the scope of this study. As a result of the analysis, Cronbach's Alpha reliability coefficient of the scale was determined as 0.84.

As a result of factor analysis, the KMO coefficient was determined as 0.864, and the Chi-Square coefficient as a result of Bartlett's Sphericity Test was determined as 1341.963. In this context, the validity and reliability of the study were considered appropriate.

As a result of the factor analysis, it was determined that all 10 items were collected under one factor and this factor explained 42.315% of the total variance. It was observed that the factor loads of the items were between 0.380 and 0.762.

Research universe and sampling

The university where the study was conducted consisted of the physicians working in the branches of surgery and internal medicine in all private hospitals in the geographical region of Silivri-Tuzla in the province of Istanbul, and patients who received health services from these physicians.

This study included 35 (20.6%) hospitals out of 170 private hospitals which participated in this study on a voluntary basis operating in Istanbul province. Since all the hospitals involved in the study were similar, they were not divided into homogeneous groups. Data were collected from 70 physicians working in surgery and internal medicine branches and 420 patients receiving health services from these hospitals on a voluntary basis. In the study, our aim was to recruit at least five patients of each physician. Although the sample size of the patients was not calculated in the study, we recruited patients at least five times the number of items on the scale for validity and reliability analyzes.

Analysis of data

The data were analyzed using Statistical Package for the Social Sciences, SPSS.24 (IBM Corp., Armonk, NY) program. Normality analysis for scale and total scores was obtained before the analysis of data was conducted via the Kolmogorov-Smirnov test. Total scores of the research scale were in the range of ±3. The difference between the mode-median-mean-cropped mean values ​​was small. The value obtained by dividing the interquartile range by the standard deviation was in the range of 1-3, and the skewness value (−0.104, p<0.002) was sufficient. The histogram-normality and box plots showed normal and nearly normal distribution. The findings obtained showed that the scale, total scores fit the assumption of normal distribution and it was decided to use parametric tests.

For data analysis, descriptive statistical tables for the sample group and scale scores were prepared. Item analysis was used in calculating reliability and validity coefficients. Independent Sample t-test was used for examining the difference between mean scores for two-category variables and one-way analysis of variance (ANOVA) was used for examining the difference between mean scores for variables with more than two categories and research scale and sub-dimension total scores.

## Results

This study was based on the assumption that all the participants in the study cohort answered honestly and based on their real situation in the "Sociodemographic Information Form", "Physician Empathy Scale" and "Patient Perception Scale on Physician Empathy".

Characteristics of recruited physicians and patients for the study

A total of 70 physicians including 35 (50.0%) internal medicine specialists and 35 (50.0%) surgeons participated in the study; 38 (54.3%) of the physicians were female and 32 (45.7%) were male. As for the age of physicians is concerned, 29 (41.4%) physicians were 40 years old or below and 41 (58.6%) physicians were aged 41 years and over. In terms of professional experience, 41 (58.6%) physicians had experience of 20 years and less and 29 (41.4%) physicians were with 21 years or more experience.

Of the total 420 patients, 210 (50.0%) patients were under treatment in the internal medicine department and 210 (50.0%) patients were treated at the surgery department. A total of 261 (62.1%) patients were female and 159 (37.9%) were male; 138 (32.9%) patients were under the age of 30 years, 123 (29.3%) patients between the ages of 31 and 40 years, 93 (22.1%) patients between the ages of 41 and 50 years, and 66 (15%) patients aged 51 years and older. When evaluated for educational level, 194 (46.2%) patients were holding university-level education and 226 (53.8%) patients were with high school or below education level; 347 (82.6%) patients were in the non-health profession group, and 73 (17.4%) patients in the health profession group. 

The age range of physicians was between 32 and 60 years with an average age of 43.04 ± 6.36 years. The range of professional experience of the physicians in the study was between 9 and 38 years, with an average of 20.09±6.40 years.

Physician empathy score was in the range of 61-121 points. The average value calculated was 97.99±14.83. Patients’ age was between 17 and 89 years, with a mean of 38.28 ± 12.86 years. The patient's perceived empathy score was in the range of 15-70 points, with an average value of 46.29±12.86.

The reliability coefficient for the Physician Empathy Scale was calculated to be 0.75, and the reliability coefficient for the Patient Perceived Empathy Scale was found to be 0.84 as shown in Table 1.

**Table 1:** Reliability Coefficients for Empathy Scales of Physicians and Patients

Scale/subdimension	Cronbach Alfa
Physician Empathy Scale	0.75
Patient Perceived Empathy Scale	0.84

A significant difference was found between the empathy score averages of physicians from the internal medicine and surgery department (t (60,444) = 4.101; p <0.001). It was observed that the average empathy score of physicians who were internal medicine specialists was significantly higher. No significant difference was found between the mean empathy scores of male and female physicians (t (68) = 1.239; p>0.05).

Empathy score averages of physicians aged 40 years or below were significantly higher than physicians of 41 years of age and above (t (68) = 2.132; p<0.05; Table 2).

**Table 2:** Investigation of the Difference Between Perceived Empathy Scale Total Scores According to Patients' Demographic Information *p<0.05; **p<0.01; *** p<0.001.

Değişken	Category	n	x̅	ss	t-F	sd	p
Outpatient clinic in the hospital	Internal medicine	210	43.18	11.88	−5.104	418	0.000^***^
	Surgery	210	49.40	13.08			
Gender	Women	261	46.03	12.84	−0.535	418	0.593
	Men	159	46.72	12.93			
Education level	High school and below	226	44.69	12.70	−2.778	418	0.006^**^
	University	194	48.16	12.84			
Profession	Health professional	347	45.45	12.58	−2.946	418	0.003^**^
	Other	73	50.29	13.52			
Age	30 years and under	138	47.12	13.51	1.465	3.416	0.223
	Between 31 and 40 years	123	46.85	12.87			
	41–50 years old	93	46.45	12.40			
	51 years and older	66	43.30	11.94			

It was observed that the empathy mean score of physicians with 20 years or less professional experience was significantly higher than those having 21 years of professional experience (t (68) = 2.141; p<0.05).

We further analyzed the difference between the total mean scores of the Perceived Empathy Scale according to the demographic information of the patients. It was observed that the average score of perceived empathy of the patients who received service from the surgery outpatient clinic was significantly higher than those who received treatment from the internal medicine department (t (418) = −5.104; p<0.001).

No significant difference was found between the perceived empathy score averages of female and male patients (t (418) = -0.535; p>0.05). We also observed that the perceived empathy score average of the patients with a university education level was significantly higher than patients with high school or below education (t (418) = −2.778; p<0.01).

No significant difference was found between the perceived empathy score averages of the patients according to age categories (F (3/416) = 1.465; p>0.05). A negative, low-level correlation coefficient of r = −0.106, significant at p<0.05 level, was observed between the patient's age and the patient's perceived empathy score. According to this result, as the patient's age increases, the empathy score perceived by the patient will decrease.

A positive, moderate correlation coefficient of r = 0.349, significant at p<0.01 level, was observed between the physician empathy score and the patient's perceived empathy score. According to this result, as the physician's empathy score increases, the patient's perceived empathy score will increase, or as the physician's empathy score decreases, the patient's perceived empathy score will also decrease as shown in Table 3.

**Table 3:** Examination of the Relationship Between Empathy Scores of Patients and Physicians *p<0.05; **p<0.01; ***p<0.001.

	Patient perceived empathy score	Physician empathy score
Patient age	−0.106^*^	0.277^*^
Physician age	−0.006	−0.199
Physician profession experience	−0.019	−0.141
Physician empathy score	0.349^**^	

A positive, low-level correlation coefficient of r = 0.277, significant at p<0.05 level, was observed between patient age and physician empathy score. According to this result, as the patient's age increases, the empathy score of the physician will decrease.

## Discussion

This study was conducted to understand the empathy of the physician towards their patients during the treatment and the perceived level of patients to the empathy shown by the physicians. Surgeons working for the private hospitals in the geographical region of Silivri-Tuzla in the province of Istanbul and patients receiving service from these hospitals were included in the study. Demographic data of the recruited cohort of physicians indicated that the majority of the surgical and internal medicine physicians were female physicians with the age of 41 years and above and professional experience of 20 years or less. A total of 49.8% of the patients included in the study received service in internal medicine and 50.2% in surgical branches. Further data indicated that the empathy levels of physicians differed statistically according to their branch of specialty. It was found that physicians working in the internal medicine units had more empathy towards their patients than physicians working in surgical units. This indicates that physicians were more in contact with patients during the diagnosis and treatment stages than surgical physicians, and thus they can reveal the problems of their patients more accurately. Our results were in concordance with the earlier published reports [7-10]. However, the type and characteristics of patients coming to a particular branch of medicine also decide the time they spend with the physicians. In the case of internal medicine, more time is allocated to the patients based on their characteristics but this does not hold true for every internal medicine branch. This could be explained with the example of a dermatologist wherein time allocated by the dermatologist for the patients is not the same as allocated by a family practice specialist to his patient. The family practice specialist has to spend a longer time.

It was found that the empathy level of physicians did not differ significantly with the gender variable, which is one of the demographic variables. Accordingly, it was also revealed that female or male physicians empathize equally with their patients. Our finding differed from the study conducted by Hojat et al. [9]. They stated that female physicians offer more emotional support messages in their approach to their patients which could be because of their more emotional sensitivity towards patients and therefore, they are able to empathize with patients more easily.

It is very important to understand, how much of what the physician conveys to his patient during the physician-patient interviews is understood by the patient as these interactive interviews form the basis of complete clinical study [11]. It would be easier for a patient working in the health sector to understand what his physician conveyed compared to patients with a non-health profession. Our data also suggested the similar fact that educated patients in the healthcare profession can establish a better understanding relationship with their physicians.

Overall data suggest that the perceived level of patients for empathy is directly correlated with empathy developed by the doctors. Understanding the importance of empathy, few points could be considered to increase the empathy level perception developed by the physicians towards them. Physicians should be more polite, patient with friendly language when interacting with stressed patients, and allow the patient to demonstrate a comfortable attitude keeping into account the gestures and facial expressions used by patients. They should never mention the estimated results in the treatment process of patients and should express the final results to the patient in an appropriate language [11]. Studies have been conducted to assess the effect of empathy in reducing stress. A study was conducted to evaluate the effect of verbal empathy and intentional touch from a bronchoscopist in reducing anxiety in 267 patients undergoing bronchoscopy. It was observed that verbal empathy and touch provided by a bronchoscopist before the procedure reduced the anxiety in those patients who had higher baseline levels of anxiety [12]. Another clinical trial conducted among breast cancer survivors and women without breast cancer showed that compassion shown by the physician in a videotape shown to them was effective in reducing anxiety among these females [13]. Socio-cultural and educational levels of the patients should not be considered while communicating with them.

Along with this medical organizations could specifically provide more training in the field of health communication during their medical education by the psychiatrists to the physicians. There should be a separate compartment for physician secretaries in the outpatient clinic rooms of physicians, and it may be appropriate for patients to communicate directly with the secretary and then contact the physician. Additional fees may be paid to physicians' salaries in line with the satisfaction of the patients they treat. Activities under the name of “public service ad” can be carried out to increase respect for society and physicians and to increase awareness on this issue.

Limitations of the study

(i) Private hospitals in the geographical region of Silivri-Tuzla, operating in the province of Istanbul, are limited to physicians working in the Surgical and Internal Medicine fields and to the patients receiving service from these physicians. (ii) The data used in the research are limited to the data collected in 2018-2019. (iii) Moreover, observations in this study are limited to the data collected through the "Physician Empathy Scale" and "Patient Perception Scale on Physician Empathy."

## Conclusions

The study concluded that physicians who conduct polyclinics in internal medicine branches have high empathy. Both male and female physicians have an equal empathy score indicating that gender does not influence the empathy shown by physicians towards their patients. Moreover, the empathy shown by physicians of less age and less professional experience was more as compared to older and more experienced physicians which might be because they are more enthusiastic about their career. The gender of the patients perceiving empathy from the physicians does not affect the perceived empathy score but as the age of patients increases, the empathy score perceived by them decreases as well as empathy score of physicians also decreases. Empathy scores of both physicians and patients' perceived empathy scores are directly correlated indicating that there should be a good coherence with what the physician is explaining to the patient and how comfortable, the patient is in understanding. There is a need for more focus on this aspect with the fact that along with keeping in consideration how physicians interact with their patients, there is also a need to provide better training to the physicians by expert psychiatrists and appreciate their efforts by various means which could encourage the practice of empathy in clinics.

But, the majority of society is examined by family physicians. Therefore, since family medicine is also an internal branch, it would be appropriate to plan a new study only for family physicians.
